# Data describing the effects of dietary bioactive agents on colonic stem cell microRNA and mRNA expression

**DOI:** 10.1016/j.dib.2015.12.026

**Published:** 2015-12-19

**Authors:** Manasvi S. Shah, Eunjoo Kim, Laurie A. Davidson, Jason M. Knight, Roger S. Zoh, Jennifer S. Goldsby, Evelyn S. Callaway, Beyian Zhou, Ivan Ivanov, Robert S. Chapkin

**Affiliations:** a Program in Integrative Nutrition & Complex Diseases, United States; b Intercollegiate Faculty of Genetics, United States; c Department of Molecular and Cellular Medicine, United States; d Department of Electrical Engineering, United States; e Department of Statistics, United States; f Department of Veterinary Physiology & Pharmacology, United States; g Center for Translational Environmental Health Research, Texas A&M University, College Station, TX, United States; h Divison of Endocrinology, Boston Children’s Hospital, United States; i Department of Pediatrics, Harvard Medical School, Boston, MA, United States

## Abstract

With the identification of Lgr5 as a definitive marker for intestinal stem cells, we used the highly novel, recently described, **Lgr5-EGFP-IRES-cre ER**^**T2**^**knock in** mouse model. Mice were injected with azoxymethane (AOM, a colon carcinogen) or saline (control) and fed a chemo-protective diet containing n-3 fatty acids and fermentable fiber (n-3 PUFA+pectin) or a control diet (n-6 PUFA + cellulose). Single cells were isolated from colonic mucosa crypts and three discrete populations of cells were collected via fluorescence activated cell sorting (FACS): Lgr5^high^ (stem cells), Lgr5^low^ (daughter cells) and Lgr5^negative^ (differentiated cells). microRNA profiling and RNA sequencing were performed from the same sample and analyzed. These data refer to ‘**Comparative effects of diet and carcinogen on microRNA expression in the stem cell niche of the mouse colonic crypt’** (Shah et al., 2016) [5].

**Specifications table**

**Table:** 

Subject area	*Biology*
More specific subject area	*Intestinal stem cells*
Type of data	*Tables*
How data was acquired	*The raw data was generated using an Illumina sequencer and was statistically analyzed and displayed in tabular format.*
Data format	*Analyzed*
Experimental factors	*The samples were collected from mice fed with either corn oil+ cellulose or fish oil+pectin. Both groups of mice were then either injected with Azoxymethane (carcinogen) or saline (control). Total = 20 mice in all.*
Experimental features	*Colonic crypts were isolated from the stem cell reporter mice and were sorted based on GFP into high, low and negative cell populations using a BD FACS Aria II cytometer/sorter (BD Biosciences). Total RNA was isolated from the sorted cells using a miRVana miRNA isolation kit. After checking the quality of the RNA, libraries were subjected to RNA sequencing and microRNA profiling.*
Data source location	*College Station, Texas, USA (30.6014*^*o*^*N, 96.3144*^*o*^*W)*
Data accessibility	*All the datasets mentioned in this manuscript will be uploaded to the Gene Expression Omnibus (GEO) (accession no.- SRP061188) in NCBI.*

**Value of the data**

• These data will serve as a basis to compare Lgr5^high^ stem cells that have been perturbed by inflammation, radiation or knockout of tumor suppressors/oncogenes.
• These data describe expression values for miRNAs in Lgr5^high^, Lgr5^low^ and Lgr5^neg^ cells. For example, if a researcher wants to know if a certain miRNA is expressed in the colonic crypt, this database will provide that information. The data set also details the location of the miRNA throughout the entire crypt.
• RNA sequencing data from Lgr5^high^, Lgr5^low^ and Lgr5^neg^ cells also provides the basis for determining the expression of different mRNAs throughout the crypt. This database will serve as a comparative platform for future studies to determine correlations between current and future datasets.

## 1 Data

The data presented in this article represent: (a) Ct values of miRNAs expressed in mouse colonic epithelial cells (Table 1), (b) differentially expressed miRNAs in Lgr5^high^ versus Lgr5^negative^ cells (i.e., stem cells vs. differentiated cells) (Table 2), (c) the effect of diet on miRNA expression in Lgr5^high^ sorted cells (Table 3), (d) the effect of carcinogen on miRNA expression in Lgr5 high sorted cells (Table 4) and (e) the effect of diet and carcinogen combination on miRNA expression in GFP^negative^ sorted cells (Table 5).

These data refer to our recently published paper ‘Comparative effects of diet and carcinogen on microRNA expression in the stem cell niche of the mouse colonic crypt’ (Shah et al., 2016) [5].

## 2 Experimental design, materials and methods

### 2.1 Experimental diets

Lgr5-EGFP-IRES-creER^T2^ mice were assigned to one of the two diet groups (fish oil / pectin or corn oil / cellulose), which differed only in the type of fat and fiber. Diets contained (g/100 g diet): dextrose, 51.00; casein, 22.40; D,L-methionine, 0.34; American Institute of Nutrition (AIN)-76 salt mix, 3.91; AIN-76 vitamin mix, 1.12; choline chloride, 0.13; pectin or cellulose, 6.00. The total fat content of each diet was 15% by weight with the n-6 PUFA diet containing 15.0 g corn oil/100 g diet (Dyets, Bethlehem, PA) and the n-3 PUFA diet containing 11.5 g fish oil/100 g diet (Omega Protein, Houston, TX) plus 3.5 g corn oil/100 g diet to prevent essential fatty acid deficiency. All diet ingredients except oils were obtained from Bio-serv (Frenchtown, NJ). To prevent the formation of oxidized lipids, diets were stored at −20 °C and provided fresh to animals every day.

### 2.2 Fluorescence activated cell sorting of colonic stem cells

Colonic crypts from individual mice were isolated as previously described Sato et al*.* [1] with minor modification. The intact colons were everted on a disposable mouse gauge needle (Instech Laboratories) and incubated with 20 mM EDTA in PBS at 37 °C for 30 min. Following transfer to chilled Ca/Mg free HBSS, colons were vigorously vortexed to release crypts. The crypts were then incubated with 50 ul of DNase (stock concentration – 20 units/ml) in 10 ml of trypsin solution and single cells were then passed through a 40 micron cell strainer. Cells were counted and resuspended to a final cell density of 2×10^6^ cells/mL. FACS (Fluorescence activated cell sorting) was then carried out to isolate the Lgr5^high^ expressing stem cells, Lgr5^low^ expressing daughter cells and Lgr5^negative^ cells isolated from the colon using a BD FACS Aria II cytometer /sorter (BD Biosciences). Cells from wild type mice were used to set the gates for sorting.

### 2.3 RNA analyses

Total RNA from Lgr5^high^, Lgr5^low^ and Lgr5^negative^ sorted cells was isolated. For this purpose, cells from individual mice from all 4 groups (total of 60 samples) were separately processed using the mirVana miRNA Isolation Kit according to manufacturer’s instructions (Ambion, Austin, TX). Expression of 368 mature miRNAs was determined using TaqMan Rodent MicroRNA A Array 2.0 (Life Technologies, Grand Island, NY) as we have previously described [2], [3]. For mRNA profiling, samples were randomized prior to RNASeq library preparation. Sequencing libraries from RNA (10 ng) were generated using the TruSeq RNA Sample Preparation kit (Illumina, San diego, CA). ERCC (Life Technologies, Grand Island, NY) was added at the appropriate level as per manufacturer instructions. The libraries were pooled and sequenced using an Illumina HiSeq 2500 at SeqWright Genomic Services (Houston, TX). Sequencing data was provided demultiplexed and aligned using STAR with default parameters [4] and referenced against *Mus musculus* (UCSC version *mm10*)**.**

### 2.4 Statistical analyses

For miRNA and mRNA profiling, two sided *t*-tests with Welch correction for unequal variance were performed on select miRNA across the specific treatment comparisons of interest. Mann–Whitney *U* nonparametric tests were also performed as a control against non-normal data and similar *P*-values were obtained. Standard error bars were plotted in order to document the variation in the population mean. *P* values <0.05 were considered to be statistically significant, and genes were selected for analysis using prior knowledge without considering *P*-values. Therefore, no multiple testing correction procedure was used. Standardized differences for the miRNAs and mRNAs were computed and a two-sample *t*-test was utilized to compare them. Small *p*-values indicated strong evidence of the hypothesized trend.

## Figures and Tables

**Table 1:** Ct values of 103 miRNAs expressed in mouse colonic epithelial cells.

**miRNA**	**Ct values**	**miRNA**	**Ct values**	**miRNA**	**Ct values**
mmu-miR-31	11.44	mmu-miR-671-3p	26.81	mmu-let-7g-	28.94
rno-miR-190b	14.92	mmu-miR-215	26.81	mmu-miR-103	28.95
mmu-miR-872	17.72	mmu-miR-151-3p	26.9	mmu-miR-320	29
mmu-miR-124	20.88	mmu-let-7b	26.96	mmu-miR-218	29.02
mmu-miR-128a	20.99	mmu-miR-203	26.97	mmu-miR-30d	29.2
mmu-miR-429	21.89	mmu-miR-484	27.02	mmu-miR-125b-5p	29.2
mmu-miR-148b	22.68	mmu-miR-29a	27.11	mmu-miR-205	29.63
mmu-miR-324-5p	22.88	mmu-miR-29b	27.24	mmu-miR-18a	29.63
mmu-miR-322	23.04	mmu-miR-340-5p	27.41	mmu-miR-195	29.68
mmu-miR-142-3p	23.43	mmu-miR-139-5p	27.43	mmu-miR-28	29.7
mmu-miR-192	23.51	mmu-miR-15b	27.43	mmu-miR-574-3p	29.83
mmu-miR-200a	23.75	mmu-miR-93	27.49	mmu-miR-101a	29.83
mmu-miR-423-5p	23.88	mmu-let-7e	27.55	mmu-miR-29c	30.01
mmu-miR-375	23.94	mmu-miR-20b	27.71	rno-miR-345-3p	30.02
mmu-miR-10b	24.03	mmu-miR-27b	28.01	mmu-miR-301b	30.12
mmu-miR-19b	24.16	mmu-miR-30a	28.04	mmu-miR-146b	30.29
mmu-miR-30c	24.31	mmu-miR-27a	28.04	mmu-miR-744	30.34
mmu-miR-92a	24.36	mmu-miR-148a	28.06	mmu-miR-331-3p	30.37
mmu-miR-191	24.43	mmu-miR-222	28.13	mmu-miR-186	30.38
mmu-miR-30b	24.75	mmu-miR-141	28.18	mmu-miR-196b	30.39
mmu-miR-24	24.78	mmu-miR-188-5p	28.19	mmu-miR-340-3p	30.44
mmu-miR-126-3p	24.79	mmu-miR-106b	28.22	mmu-miR-301a	30.45
mmu-miR-17	24.91	mmu-miR-19a	28.23	mmu-miR-130b	30.49
mmu-miR-194	24.92	mmu-let-7d	28.23	mmu-miR-193b	30.68
mmu-miR-200b	24.99	mmu-miR-25	28.24	mmu-miR-155	30.77
mmu-miR-34b-3p	25.05	rno-miR-196c	28.46	mmu-miR-152	30.8
mmu-miR-20a	25.16	mmu-miR-130a	28.46	mmu-miR-23b	30.98
mmu-miR-106a	25.16	mmu-miR-100	28.5	mmu-miR-183	31.39
mmu-miR-200c	25.38	mmu-miR-30e	28.57	mmu-miR-125a-5p	31.84
mmu-let-7c	25.74	mmu-miR-182	28.57	mmu-miR-181a	31.94
mmu-miR-16	25.98	mmu-miR-328	28.59	mmu-miR-181c	34.76
mmu-miR-10a	26.11	mmu-let-7i	28.68		
mmu-miR-145	26.32	mmu-miR-26b	28.84		
mmu-miR-26a	26.33	mmu-miR-140	28.86		
mmu-miR-21	26.48	mmu-miR-146a	28.87		
mmu-miR-99a	26.67	mmu-miR-224	28.92		

Expression of miRNAs was quantified by reverse transcription using miRNA-specific primers followed by real-time PCR TaqMan low-density array analysis. Ct values represent means of 60 samples, mmu, mouse; rno, rat; Ct, cross threshold.

**Table 2:** Differentially expressed miRNAs in Lgr5^high^ versus Lgr5^negative^ cells.

Up-regulated in Lgr5^high^		*P*-value	Down-regulated in Lgr5^high^		*P*-value
miRNA	Expression ratio Lgr5^high^/ Lgr5^negative^	miRNA	Expression ratio Lgr5^high^/ Lgr5^negative^
mmu-miR-342-3p	2.64	0.012	mmu-miR-652	0.32	0.000
mmu-miR-671-3p	2.29	0.008	mmu-miR-145	0.40	0.009
rno-miR-345-3p	2.14	0.022	mmu-miR-27a	0.42	0.000
rno-miR-190b	1.95	0.018	mmu-miR-215	0.42	0.000
mmu-miR-155	1.90	0.033	mmu-miR-532-5p	0.50	0.040
mmu-miR-191	1.81	0.001	mmu-miR-7b	0.60	0.027
mmu-miR-20b	1.68	0.011	mmu-miR-21	0.62	0.024
mmu-miR-17	1.68	0.000	mmu-miR-30d	0.62	0.017
mmu-miR-125a-5p	1.58	0.036	rno-miR-224	0.62	0.018
mmu-miR-186	1.58	0.006	mmu-miR-30a	0.66	0.000
mmu-miR-218	1.44	0.003	mmu-miR-200b	0.69	0.013
mmu-miR-10a	1.30	0.047	mmu-miR-203	0.76	0.003
mmu-miR-92a	1.29	0.018			
mmu-miR-200a	1.27	0.031			

Expression of miRNAs were quantified as described in Table 1. GFP^high^ stem cells (*n*=20, pooled samples); Lgr5^negative^ cells (*n*=20, pooled samples). Only miRNAs with *P*<0.05 are shown. mmu, mouse; rno, rat.

**Table 3:** Effect of carcinogen on miRNA expression in Lgr5^high^ sorted cells.

A.		
miRNA	Expression ratio AOM-Lgr5^high^ /Saline-Lgr5^high^	*P*-value
mmu-miR-532-3p	2.61	0.020
rno-miR-196c	1.66	0.008
mmu-miR-331-3p	1.45	0.032
mmu-miR-92a	1.41	0.042
mmu-miR-100	0.19	0.050
mmu-miR-124	0.25	0.021
B.		
miRNA	Expression ratio AOM-Lgr5^neg^ /Saline-Lgr5^neg^	*P*-value

mmu-let-7e	1.97	0.008
mmu-miR-18a	1.84	0.050
mmu-miR-20b	1.59	0.029
mmu-miR-101a	1.52	0.045
mmu-let-7i	1.45	0.039
mmu-miR-375	1.26	0.046
mmu-miR-224	0.65	0.030
mmu-miR-193b	0.65	0.045
mmu-miR-10a	0.62	0.008

miRNA expression was quantified as described in Table 1. AOM, azoxymethane (*n*=20); saline (*n*=20); Lgr5^high^, stem cells; Lgr5^neg^, non-stem cells. Only miRNAs with *P*<0.05 are shown.

**Table 4:** Effect of diet on miRNA expression in Lgr5^high^ sorted cells.

A. Carcinogen		
miRNA	Expression ratio CCA-Lgr5^high^ /FPA-Lgr5^high^	*P*-value
miR-21	2.2	0.030
miR-26b	2.0	0.010
miR-200a	1.8	0.000
miR-10a	1.7	0.040
miR-26a	1.7	0.020
miR-29c	1.6	0.010
miR-30c	1.5	0.040
miR-203	1.5	0.040
miR-30a	1.4	0.020
miR-19b	1.3	0.040
miR-181a	0.6	0.030
miR-34b-3p	0.1	0.000
B. Saline		
miRNA	Expression ratio CCS-Lgr5^high^/FPS-Lgr5^high^	*P*-value

mmu-miR-188-5p	5.3	0.027
mmu-miR-218	0.6	0.016
mmu-miR-125a-5p	0.5	0.047
mmu-miR-574-3p	0.5	0.028
mmu-miR-200c	0.5	0.047
mmu-miR-222	0.4	0.016
mmu-miR-429	0.3	0.034
mmu-miR-106a	0.1	0.047

Expression of miRNAs was quantified as described in Table 1. CCA, Corn oil+cellulose+azoxymethane (AOM) (*n*=5); FPA, Fish oil+pectin+AOM (*n*=5); CCS, Corn oil+cellulose+saline (*n*=5); FPS, Fish oil+pectin+saline (*n*=5); Lgr5^high^, stem cells. Only miRNAs with *P* ≤0.05 are shown.

**Table 5:** Effect of diet and carcinogen combination on miRNA expression in GFP^negative^ sorted cells.

miRNA	Expression ratio CCA- Lgr5^neg^/FPA- Lgr5^neg^	*P*-value
miR-29b	3.8	0.008
let-7e	2.1	0.004
Let-7c	1.3	0.048
miR-19b	0.6	0.029
miR-484	0.5	0.019
miR-19a	0.4	0.034
miRNA	Expression ratio CCA- Lgr5^neg^/FPA- Lgr5^neg^	*P*-value
miR-29b	3.8	0.008
let-7e	2.1	0.004
Let-7c	1.3	0.048
miR-19b	0.6	0.029
miR-484	0.5	0.019
miR-19a	0.4	0.034

Expression of miRNAs was quantified as described in Table 1. CCA, Corn oil+cellulose+azoxymethane (AOM) (*n*=5); FPA, Fish oil+pectin+AOM (*n*=5); Lgr5^neg^, differentiated cells. Only miRNAs with *P*≤0.05 are shown.

## References

[1] Sato T., Vries R.G., Snippert H.J., van de Wetering M., Barker N., Stange D.E. (2009). Single Lgr5 stem cells build crypt-villus structures in vitro without a mesenchymal niche. Nature.

[2] Davidson L.A., Wang N., Shah M.S., Lupton J.R., Ivanov I., Chapkin R.S. (2009). n-3 Polyunsaturated fatty acids modulate carcinogen-directed non-coding microRNA signatures in rat colon. Carcinogenesis.

[3] Shah M.S., Schwartz S.L., Zhao C., Davidson L.A., Zhou B., Lupton J.R. (2011). Integrated microRNA and mRNA expression profiling in a rat colon carcinogenesis model: effect of a chemo-protective diet. Physiol. Genom..

[4] Dobin A., Davis C.A., Schlesinger F., Drenkow J., Zaleski C., Jha S. (2013). STAR: ultrafast universal RNA-seq aligner. Bioinformatics.

[5] Shah M.S., Kim E., Davidson L.A., Knight J.M., Zoh R.S., Golsdby J.S. (2016). Comparative effects of diet and carcinogen on microRNA expression in the stem cell niche of the mouse colonic crypt. Biochim. Biophys. Acta.

